# Dr. Jackson on the Epidemic Yellow Fever of Cadiz and the South Coasts of Spain

**Published:** 1821-12-01

**Authors:** 


*Remarks on the Epidemic Yellow Fever which has appeared at intervals, on the South Coasts of Spain, since the year* 1800. By Robert Jackson, M.D. One vol. 8vo, pp. 207. London, 1821.

Prone as the world is, in general, to vice, there are numerous and splendid examples of virtue in every age and country—and beset as the medical profession is by selfishness, knavery and charlatanism, there is, thank Heaven, a great deal of honour, integrity, disinterestedness—we had almost said *devotion* to the advance of knowledge and the interests of humanity, scattered through its members. We see indeed, with feelings of humiliation for poor human nature, too many men, whose education ought to have elevated their views, sacrifice all the nobler sentiments of humanity at the shrine of SELF, or cast off all sense of shame, and turn the credulity of mankind into a source of lucre to themselves, totally regardless of the contempt or even abhorrence of their brethren. But, on the other hand, the profession exhibits numerous examples of men devoting their time, their talents, their wealth, and what is more than all, their HEALTH, or even LIFE, to the welfare of society at large, or to a posterity who can only know them by name. We do not wonder so much at these instances of philanthropy in *youth*, whose warm blood nurtures generous sentiments, feeds enthusiastic feelings, and engenders those aspirations after ideal beatitude, which time and experience too often prove to be unattainable by mortals. But when we see this philanthropy grow with man's growth, and strengthen with his strength—when we see it as fervid at sixty-five as at twenty-five, unchilled by hoary age, unchanged by length of years, it commands the respect of the most unprincipled, and excites the admiration of the most virtuous portions of society.

The venerable author of the work before us has sustained a character of this description, from the earliest period of his professional career, down to the present moment. Whether serving amid the foggy marshes of Flanders, the burning defiles of St. Domingo, the dreary savanhas of America, or the romantic vallies of Spain, Dr. Jackson was actuated by but one ruling passion, one predominant impulse—the acquisition of useful knowledge for the promotion of MEDICAL SCIENCE, not the advancement of SELF. This thirst after knowledge, this true philosophy, has not deserted him in his old age, for, *qualis ab incepto*, he lately left the comforts and quietude of a country retirement, so congenial to declining life, to wander among scenes of pestilence and death in a foreign land—and that without the chance of remuneration, but the certainty of loss as well as danger and fatigue.

The Cadiz fever of 1819 having excited our author's curiosity to ascertain its true nature, he applied to Government for a passage and credentials to the Spanish authorities, without any other condition or exemption from expense.

The passage and introductions were granted, but not till after a lapse of time which rendered it improbable he should that year see the epidemic, excepting in its decline. He embarked, however, in the beginning, and landed at Gibraltar towards the end, of December. There he was detained nearly two months, in consequence of a military insurrection at Cadiz. There being now no hopes of seeing the epidemic of that season, Dr. Jackson determined on a journey to the Levant, at his own expense, partly in the desire of seeing Greece, a country in which he had lived in idea the greater part of his life, and partly in the hope of obtaining information respecting the diseases (particularly plague) of the Mediterranean shores. He therefore visited Malta, Constantinople, Smyrna, the Islands of the Archipelago, Athens, and the Morea, returning to Gibraltar in the end of July 1820, from whence he proceeded to Cadiz, where he arrived on the very day that the epidemic was officially announced.

In this expedition (so different in its object from most other expeditions) our able author was accompanied by Mr. O Hallaran, assistant surgeon of the 64th regiment, a gentleman "who had courage to look danger in the face in whatever shape it might present—zealous in the pursuit of professional knowledge, and of an ardency of temper which committed him wholly to the duty which he undertook." This gentleman was of infinite service to our author in *post mortem* researches, as well as therapeutical labours.

The first chapter of the work contains an animated sketch of the medical topography of Cadiz and its environs, of which we can present but a very few features. The province of Andalusia is of irregular surface, the scenery being, in many places, beautifully picturesque. The elevated part of the province is generally dry, of a light unproductive soil, and covered with heath or brushwood. The extensive level plains are nearly in a state of bog in the rainy season; and the soil being loose and light, the water penetrates deep into it, and rises again in copious vapour during the great heats of summer. It is well known that exhalations from the earth cause, or at least carry with them the cause of, intermittent fevers. Accordingly we find that the people of Andalusia who live on the plains, or on eminences near the plains, are "much harrassed by agues."

"The type sometimes assumes the quartan form; sometimes it appears as distinct remittent; and sometimes it sinks into obscure remittent or gastric. From gastric there is an easy transition to continued or contagious typhus; and sometimes, under epidemic influences, a form of disease not unlike that which has obtained the name of yellow fever prevails to some extent." P. 2.

Cadiz itself occupies a peninsular point nearly level with the sea, the houses being erected on shelves of rock, at one place, on alluvial sand at another—of course, many of them stand over a bed of water. The confined site of Cadiz has induced the inhabitants to build the houses very high, and make the streets very narrow—the *former*, however, being good, and the *latter* kept very clean—" insomuch that this city, crowded as it is with population, rarely, if ever, presents any thing that is offensive to the eye, or that can be supposed to be noxious to health." 4. The Isla, contiguous to Cadiz, is of considerable extent, and mostly a swamp intersected by numerous sluice canals. Yet it is not unhealthy, which may fairly be attributed to the strong salt water forming the moisture of the soil. Topographical sketches follow of Puerto Real—Puerto de Santa Maria—and Xerez, for which we must refer to the volume itself.

The second chapter is on a very important subject—the introduction (if really introduced) of the yellow fever into Spain, and its real or supposed propagation by what is termed contagion. The facts and arguments brought forward by Aregula, or still entertained in that country, are dispassionately examined and candidly weighed by our author, aided by all the information he could gain on the spot; and this discussion we recommend to the serious attention of the profession, hoping as we do, that it may lead to important conclusions, medical and commercial. Mean time, we shall endeavour to present our readers with some of the results to which our author has come, from the details alluded to.

1. The importation of the disease from a foreign country is still credited by the authorities and mass of people in Spain, though, Dr. J. thinks, it has never been proved by evidence, or even brought to reasonable probability—the events of the year 1820 stripping the assumption of every claim to credence, as no attempt has been made to trace the disease, in that instance, to foreign origin.

2. The belief universally obtains through Spain that the disease is personally contagious; that is, capable of propagation from individual to individual, by contact or proximity. Yet our author thinks that this opinion, confidently as it is maintained, is invalidated by authentic records and facts.

" In proof of the assertion, an authority is here adduced which may be thought to be valid, because it is admitted by the persons who report it against the dictate of their preposessions. In the year 1800, when upwards of ten thousand souls died at Xerez de la Frontera, sixty persons were employed to bury the dead. The burriers entered the houses where the dead lay, took the bodies in their arms, often it is presumed in a loathsome state, put them into the carts in heaps, and drove them to the place of interment. None of the buriers were infected. They were said to have been under the influence of liquor on most occasions; and, as such, were supposed to be comparatively less susceptible of the impressions of contagion than they otherwise could have been. The supposition is of some weight; it is not conclusive of the inference that has been drawn from it. It is known from other experience, that if strong contagion exist in the clothes or coverings of the dead, (and had it existed at all it must have been strong in the present case,) the power of wine is only a feeble protection." 38.

A somewhat similar event happened in the year 1819 at St. Lucar. The buriers of the dead having shamefully abused their office, the friars of the Franciscan convent volunteered the interment themselves.

"The offer was accepted; and the friars, in entering on the duty which they had thus imposed upon themselves, found the majority of the houses or sick apartments deserted, the bodies of the dead lying in various postures upon beds, or on the floor. They wrapped them in sheets, or such other covering as presented itself in the apartment, carried them to the bier in their arms, and afterwards in the bier on their shoulders to the grave. No one of this meritorious band was attacked by the disease, although five of them were supposed to be susceptible of it as not having had it at a former time." P. 39.

In corroboration of the present argument, Dr. Jackson states that washers and others who handle the impure bedding and body-linen of the sick or dead, are not, from the best information that can be obtained, more liable than others to the attacks of this disease. The contrary is the case where typhus has prevailed.

3. From the facts here stated, our author concludes "that the contagion of yellow fever does not attach itself to clothing, or other substance that has been in contact with the diseased subject."

"The disease appears suddenly and spreads rapidly as a pestilence on many occasions; but it does not spread except in epidemic atmospheres. This last is a point of high interest to the community. It is not denied by any one, at least it is asserted by many creditable witnesses, that multitudes of persons, who have removed from an infected circle, to a circle exempt from infection, either under disease or with the cause of disease so forward in preparation for explosion, that the malady, actually exploding with its genuine characteristics in a short time after the removal, has run its regular course, and terminated fatally or otherwise after a certain duration, without communicating any thing hurtful to the most assiduous of the attendants. Examples of the fact are so frequent, both in Spain and other countries where yellow fever is known, that it is impossible to refuse assent to the inference that the disease, if actually contagious, is contagious, so as to propagate its kind in epidemic atmospheres only. The simple disease is not sufficient; and, if this be admitted, the conclusion is direct that yellow fever is not exportable, unless the atmosphere of the epidemic circle be also exported in the hold of a ship or otherwise; a position which is absurd, and which directly negatives the necessity or utility of quarantine restriction." 41.

4. Yellow fever often arises after intercourse or association with the infected—it also arises where it is impossible to trace intercourse or even suspect it, as happened in 1820, when the fever appeared at Cadiz and Xerez, where there was no visible source of infection.

"It is admitted, even by those who believe the doctrine of contagion in all its extent, that those who seclude themselves fall down nearly in the same proportion as those who walk the streets; and, as the fact is distinct and often verified in experience, it furnishes conclusive evidence that yellow fever has another cause, at least may be called into existence by another cause than the direct application of personal contagion; consequently that seclusion does not hold out a warrant of security." 43.

But although the facts here stated appear decisive against the yellow fever of Andalusia being a personally contagious disease, yet, as Dr. Jackson properly remarks, a concurrence of contingent causes may produce a very imposing case of contagion, if things are superficially observed. The yellow fever, for instance, during an epidemic influence, often strikes like a pestilence by the mere concourse of people in a close place, and, "if a mass of sick persons be collected into an hospital during the epidemic season, the common emanations from the sick bodies, whether saturated with contagious particles or not, often act offensively on those who enter the circle, and often appear to be the cause of the explosion of a disease which, without such accessory or changed condition of the medium in which man lives, would have probably remained dormant for a time, and perhaps for ever."

The instances indeed of persons who have lived, in apparent good health, in simple epidemic atmospheres, and who have become sick soon after entering a crowded assembly or hospital, are so numerous and well marked, that they stagger the opinion here contended for of the non-contagious nature of yellow fever. Dr. Jackson, with that candour which belongs to true philosophy, relates many instances calculated, at first sight, to make against his own principle, of which we shall notice one or two.

"An English gentleman, of the medical profession, who had been resident in Cadiz for some months, and who was desirous to look at the reigning epidemic with his own eyes, went occasionally into the hospitals where persons ill of that malady were collected. He spent more time than usual one day in examining minutely into the condition of a person who appeared to him to be extremely ill. Next morning about eight o'clock, and about sixteen hours after he had left the hospital, he was seized with symptoms of severe indisposition, such as appeared to himself to be of very unusual kind. The indisposition in fact soon declared itself to be the prevailing epidemic; and, according to his own account, it was analogous in its symptoms to the case that had so particularly attracted his attention in the hospital." 45.

Here, Dr. J. observes, is a *prima facie* case of contagion; yet it is not conclusive. It must be recollected that the gentleman in question lived in the epidemic atmosphere of Cadiz, and traversed without reserve every part of the town. He might thus be fairly supposed to have imbibed the seeds of the disease from an unknown source.

"But there are grounds to believe that his visit to the hospital accelerated its appearance; and that the impression made by the circumstances of the patient, to whom his attention was principally directed, tended to modify the form of the morbid act when it did appear." 45.

A novice in the hospital of San Juan de Dios had been confined with something like mumps for a few days, but had returned to duty, and on the day on which he resumed it, he looked into the dead room, where a person who had died of one of the worst forms of yellow fever was under dissection. He was seized with fever in the night, and it extended to the 17th day. Another of the novices, who had been most assiduous in his attentions to the sick, and who assisted at dissections, was taken ill about the same time; but being treated in a very different manner from the former, he was convalescent on the third day. These cases will be considered by many as arising from the direct agency of contagion.

"The author is disposed to believe that the explosion here alluded to was merely contingent, whether occasioned by immersion into an atmosphere less pure than the common atmosphere, or by impression through the eye on the sensibilities of a body predisposed." 46.

We are, of course, totally ignorant of the nature of these epidemic influences in the air; neither do we know the precise morbid state of air in crowded hospitals—whether disease is favoured by things added to, or subtracted from it. But the following facts we know, for we have often felt them.

"Persons of every habit, but more especially persons of susceptible habit, who enter into the apartments of those who are ill of the epidemic fever, rarely fail to experience unpleasant sensations at stomach, viz. distention and irksomeness, not unfrequently uneasiness in the bowels, suspension, or change in the natural functions, head-ache, heat, pain of the eyes, thirst, white tongue, disturbed sleep, and dreaming amounting to reverie. These beginnings of the morbid act are local; and, as such, they are for the most part removable by the prompt application of remedies that act locally; that is, by emetics, purgatives, or others which produce decided changes in the secreting surfaces of the alimentary canal. It is presumptively by means of these remedies that Mr. O'Halloran, the gentleman who accompanied me to Cadiz, and who in a manner domesticated with the sick at Xerez, warded off the attacks of formal fever. He was almost constantly under a greater or less degree of indisposition; and I was indisposed myself on various occasions, never in health, though my visits to the sick were desultory and comparatively few. It is not said that the impressions, which produced indisposition on these occasions, were impressions from the cause of yellow fever: it is evident that the general atmosphere was epidemic; and it was probable that the atmosphere of the sick ward was so in a higher degree than elsewhere; or, if not so, that the diseased act was there suffered to explode with more facility, in consequence of the diminished coercive energy of the atmosphere which filled the sick apartments." 49.

5. The last subject of this part of the work is, the question of liability to second attacks. The author saw or heard of not fewer than twenty persons under the epidemic of 1820, who had medical certificates, and actually believed themselves that they had the malady at a former period. It is not possible, of course, for a person who has not seen this epidemic at different periods to determine this question by the evidence of his own senses; if there be any faith in the observations of medical men, or any confidence in the experience of those who have undergone the disease, our author thinks that the complete non-liability is disproved. Yet, while the rule of exemption is not absolute, our author's experience, (which coincides with that of the best observers,) goes to shew that persons who have sustained one attack either of the concentrated yellow fever of the West Indies, or of Andalusia, are little liable to suffer from it again—at least in the same degree of intensity, so long as they remain in the same place.

The third chapter is on the name, nature, and diagnostic of the disease usually called yellow fever. To the terms "typhus icterodes," "yellow fever," "exanthema internum contagiosum," &c. as applied by the Spanish physicians, our author makes valid objections; but, as he himself acknowledges, it is much easier to point out impropriety than to say what is right. It is not possible, he thinks, to offer a proper name to a malady where there is nothing specifically distinct in character to discriminate it from all others. The disease is a formidable one, but cannot perhaps be properly termed a new one. All circumstances considered, it is not possible, Dr. J. thinks, to come to any other conclusion than that it is the endemic of the place, rendered epidemic at particular but uncertain periods, by the operation of causes to us totally unknown at present. On this account our author modestly, but wisely, we think, declines giving the disease a name.

Our author makes a great number of highly interesting observations on the nature of epidemic influence, of which we shall be able to take but a very cursory glance. He considers, and justly so, the atmosphere *in* which we live, as a product of the earth *on* which we live; and thinks it very reasonable to suppose that the epidemic influence, whatever it is, is "the product of a temporary local derangement in the bowels of the earth, subversive of the order of movement among the materials which manufacture a healthy atmosphere." In this, as our readers know, we perfectly agree with Dr. Jackson, and with Sydenham himself, whose theory was far more probable and natural than any which has been substituted since.

Whether the morbid action arises from something that over-excites the system, or from some defect of what maintains the healthy movements therein, we cannot say; but the act itself, Dr. Jackson thinks, "has evidently some analogy with the effect of electrical influence, both in the manner in which it strikes the subject at first, and in the manner in which the disease produced by it proceeds to its termination." The epidemic aura is prejudicial to many other animals as well as man.

The yellow fever of Andalusia is cognizable, at first view, by the experienced eye; but the traits which discriminate it are not entirely communicable by words. Nevertheless, as it is important that the opinion be formed within the first twelve hours, Dr. J. endeavours to sketch out its most characteristic features in that early period of its course. "If the physician, he observes, remain undecided until yellowness or black vomiting supervene, it is of little consequence whether he decide or not." The eye, so peculiarly calculated to exhibit the state of the mind, is not less indicative of the state of the body in certain diseases. We shall give Dr. Jackson's observations on this subject in his own words.

"The *eye* of persons attacked with the yellow fever of Andalusia is peculiar. It is sometimes inflamed, muddy, and confused, as if had been exposed to the smoke of green wood; it is sometimes pearly white, vacant, inanimate, or like the eye of an idiot, sometimes irritable and intolerant of light: it is not cheerful and brilliant as it sometimes is at the commencement of contagious fever. These marks are striking, but they are not diagnostic. The diagnostic consists in a certain indescribable glistening suffusion. The character is not communicable to words; the impression is notwithstanding strong on the mind of those who observe, and it is cognizable at the earliest period of the existence of the disease. Besides general aspect and expression, the ball of the eye undergoes more or less of change in its coats and substance at an early period; and the appearances are so peculiar, through the whole of the after progress, as to be considered with reason as the surest index of the issue. It sometimes becomes yellow as in jaundice, sometimes fixed and glassy as if the humours were artificially congealed, and the moving power in a state of balanced action; sometimes it is lurid, the expression dull, the colour of the white dusky yellow, not unlike the colour of rancid tallow:—the glistening suffusion is still observable and still characteristic." 61.

The *countenance* also exhibits a discriminative aspect to the experienced; but it is wholly impossible to pourtray its characters in words. It is not only always devoid of the natural expression of the countenance in health, but in fevers of common character. "It is such, in fact, as indicates strongly that an unknown cause of force has constricted, and in a manner enchained the usual play of animal life."

The *invasion* of yellow fever is frequently, but not always, sudden—sometimes rapid as a stroke of lightning, then usually acting principally on the head. The first and most prominent action of the disease presents an appearance of general *constriction* throughout the capillary system. The secretions are diminished or suspended—the sensations deranged or perverted. This sense of general constriction, our author thinks, is a strong criterion of the existence of yellow fever, and he recommends the physician to particularly attend to it. But we cannot at all do justice to this or any other chapter in the work, so pregnant is every page with thought and observation, undiluted with useless verbiage. We can merely skim the surface for specimens, rather than attempt to convey a connected idea of the sense of the work.

The fourth chapter enters into a more particular history of the yellow fever, as it appeared at Cadiz and Xerez in 1820. This is a very long and important chapter, occupying nearly sixty pages of letter press. It is principally taken up in describing the symptomatology of the fever as affecting different constitutions, which he divides into the sanguineous, phlegmatic, serous, and gangrenous. These delineations are most minute, and shew the hand of a master. They must be studied in the original, as they will afford ample food for reflection in the minds of the pathologist, physician, and physiologist. We cannot attempt an analysis of them, but we shall present some extracts from Dr. Jackson's recapitulation of these historical descriptions. Thus recapitulating the symptomatology of that form of fever which is manifested in the sanguine temperament, Dr. Jackson states as follows :—

"1. The yellow fever of Andalusia manifested the principal force of its action on the organs of the great circulation on some occasions, but not in many comparatively. The action of the heart and arteries was here strong, even violent and tumultuous, occasionally more prominent on one part than another. Where the excited act was what may be called general in the system, the favourable termination was effected through sweat and hypostatic urine; the unfavourable, by quiescence from struggle as an effect of inability. The termination of the prominently local act was effected by effusion or deposition of purulent secretion in the part; the final issue was favourable or unfavourable according to circumstances of contingence." P. 114.

*Necrotomy.* The appearances on dissection vary according to the duration of the disease, and the prominent character of its symptoms. In the rapid forms, effusions of blood were sometimes found on the surface of the brain from ruptured vessels, without mark of regular inflammation. In the protracted forms, the membranes, and even substance of the brain appeared to have sustained more inflammatory action, the ventricles being generally filled with bloody serum, and the base of the brain deluged with it. "The mucous membrane of the stomach, particularly about the cardiac orifice, more or less inflamed in almost every case—in some almost gangrened. The internal surface of the intestines corresponding with that of the stomach. The liver generally turgid and increased in size—"sometimes gorged with blood in a manner rotten"—the gall bladder filled with thick, tar-like bile. No particular appearance in the other viscera.

"2. *Phlegmatic Temperament.* A form of yellow fever of restrained or bridled vascular action, impaired power of absorption, disposition to agglutination between contiguous surfaces, and stagnation of the moving fluids in particular organs, the ostensible act manifested on the system of lympho-mucous secretions, was frequent both at Cadiz and Xerez in the year 1820, but particularly at the latter during the author's sojourn at that place. The pulse, after the first tumults of invasion were past, rarely exceeded eighty strokes in a minute, sometimes not more than sixty or seventy. The strokes were equal in time as the strokes of a clock, open and free as superficially observed—not elastic and buoyant; they were such in fact as could scarcely be said to be febrile. The skin was soft, flaccid, pasty, often moist or damp. The heat was for the most part diffused generally; and it was not often much above or below the heat of health in actual measure; sometimes however the surface was cold as marble, similar to what occasionally occurs in cholera. The lips were sometimes moist and rosy, sometimes dry and pale as if from suppressed capillary circulation. The countenance was generally pale, fixed, and statue-like; sometimes it presented circumscribed flushings on the cheek bones, but without corresponding signs of animation. The eye gave an impression of stiffness and irksomeness as it moved in the socket: it was dull and torpid, inanimate and often of a pearly white, vacant in expression, and veiled as it were by a glistening suffusion that cannot be easily, if at all, described in words. The tongue was sometimes foul and coated; sometimes rough and dry—with urgent thirst; sometimes not much changed from natural. Nausea, eructation, distention, and impatience of pressure at the epigastrium were often distressing. Nausea was an early symptom; it was sometimes accompanied by peculiar indescribable sensations at the upper orifice of the stomach, and followed in most instances by vomiting of the liquids which had been recently drank, rendered ropy by morbid secretions of a dirty colour—rarely, if ever, bitter, yellow, green, or what is called bilious. On the third day in some, in others, not until the fifth, a dirty coloured fluid, interspersed with flaky substance, was thrown up in quantity—dark and muddy like turbid coffee, sometimes more intensely black like the juices of the cuttle-fish. Head-ache was often severe during the tumults of invasion; it became moderate, or disappeared as the disease proceeded to its termination. Starts of delirium occurred sometimes: torpor of the intellectual faculty, viz. a want of power to command thought, or a species of forgetfulness, like absence, was common to all. Pains and spasms in the bowels occurred often during the early period: obstinate constipation accompanied with a sense of constriction, and, at a later period, a purging of a watery, dirty, black fluid, sometimes intermixed with villous flakes and granulated substances like grains of glazed gunpowder, might be said in a manner to characterize this form of yellow fever. The bowels resisted the stimulation of the strongest purgative on some occasions; or, if they yielded through excess of force, a sense of constriction was still felt in the tract of the canal, and no essential relief was obtained from the partial evacuation. The pulse, which could scarcely be said to be febrile after the second day of the disease, became weak, small, frequent comparatively, and sometimes so sunk as to be scarcely perceptible at the latter periods. Death approached at a rapid pace in many—often without tumult or commotion;—it was precipitated by convulsion in some.—The history now given, is the history of the common routine of the unfortunate course; a course, which appears to be sometimes suspended, about the fifth or seventh day, by a stream of life thrown into the system at those times in a manner that cannot be explained, but that tends, by the new action produced, to avert death. The circulation, from drawling and sluggish, becomes buoyant and active; the tongue assumes a white and furred appearance; in short, a new train of febrile movement takes place, runs a given course, and terminates, after a short duration, sometimes favourably, sometimes otherwise. Besides revival from a forlorn condition, by what may be called an extra infusion of life into the system, certain sensations of pain and constriction, which are sometimes prominent among the symptoms of yellow fever, give way suddenly on some occasions; the natural functions resume their course in a calm manner, and health is gradually restored to its pristine state without ostensible marks of commotion or crisis." 117.

*Necrotomy.* When this form of the fever also runs its course rapidly, and terminates in twenty-four hours, the brain exhibits turgescence of vessels, preternatural firmness of the encephalon itself, effusion of water in the ventricles—not seldom at the base of the brain or in the theca vertebralis. If the course be protracted to the fourth, fifth, sixth, seventh, or later day, "the liver and spleen are often gorged with black blood, so as to be perfectly rotten; and patches of gangrene are often observed at different points in the intestinal canal, without marks of preceding inflammation." Contractions of the intestines and even intus-susceptions were not uncommon. The stomach and intestines generally contained more or less of coffee-coloured fluids—the inner coat of both organs often loose in some places, and floating in the liquid. It is curious, that "when the fever ceases, the figure is plump and round, as if there had been a complete restriction on the organs of waste or expenditure during its continuance."

The above form of fever was frequent, but that to which we are now coming was the dominant, formidable, and most common form of all, both at Cadiz and Xerez, in 1820. We shall present Dr. Jackson's recapitulatory sketch, as the best analysis of the regular and detailed description which precedes.

"3. The third form of yellow fever was the most common form in the year 1820, both at Cadiz and Xerez; in fact more than equal in amount to both the others. The morbid act was manifested principally on the system of serous secretions—external and internal. The system of the great circulation is necessarily implicated in every febrile process; but its action was here rarely excited to excess; that is, the pulse was not strong, vigorous, and expanding, such as aims at removing impediments by force: it was often frequent, sharp, and quick, more or less irritated, but apparently repressed from developing itself freely by strong capillary constriction. The skin was dry from the beginning; and it generally continued dry throughout. The lips were dry, often arid and parched; sometimes pale and bloodless as if the capillaries were obstinately constricted. The countenance was sharp and peevish, more or less contracted—in contradistinction to placid, expanded, and serene. The eye glistened and twinkled, often sparkled like the eye of a cat in a dark room, particularly at an advanced period. The white of the cornea usually became of a faint lemon colour, or dingy yellow, after the third or fourth day. The heat of the skin was generally higher than natural; sometimes acrid and pungent in an extreme degree. The texture of the skin was closely constricted, little susceptible of the stimulation of blisters, oftener seared than vesicated by their application, or, if vesicated, the parts underneath dried and shrivelled suddenly, so as to resemble dead hide rather than the skin of a living man. The disease, as acting on the sero-mucous base, appeared to have great latitude in degree of force. The constriction of the capillaries was sometimes close, so as almost to deprive the skin of its animation; sometimes it was moderate, so that life circulated through all its parts with buoyancy. The symptoms disappeared imperceptibly on some occasions in the latter case; sometimes perceptibly by an obscure crisis on the third, fifth, or seventh day. But, though this happened sometimes, there was no sure dependence on its occurrence:—the most experienced could not calculate with certainty. The countenance and surface of the body, which were animated, and as it were buoyant with life during the first and second day, suddenly became dry, shrivelled, and withered like the blighted leaf of a plant, sometimes at the commencement of the third, sometimes of the fourth, and oftener perhaps at the commencement of the fifth day. The withering act was instant, so as to be regarded as the act, not the effect of the paroxysm. The stomach, which was disagreeably affected from the beginning of the disease, began at a certain point, viz. commencement of the retrograde course, to eject a flaky liquid of a dark colour, resembling muddy coffee or the juices of the cuttle fish. The evacuations by stool were of similar appearance with what was ejected by the mouth, viz. copious, cold, insipid, watery, and black. Numerous shreds of the villous coat of the intestines floated in the liquid; and grains of a hard substance and black colour were sometimes found at the bottom of the stool-pan. The intellect, which was affected occasionally in the early stage, but rarely violently or permanently affected, was more or less disturbed at the later periods.—Spasms and convulsions sometimes ushered in death." 119.

Before proceeding farther we may be permitted to make a few reflections on what is past. In the first place, it is evident that our venerable and intelligent author has no particular pathological theory to support—and if he had, few would suspect *him* of colouring facts to suit the hue of an hypothesis. In the second place, it is sufficiently manifest, from the faithful histories abovementioned, that the phenomena of fever (whether from aerial, terrestrial, or animal effluvia) do not *spring* from inflammation or any thing analogous to inflammation, in this or that organ; for we see the disease, when violent, destroy the patient before any trace of inflammation becomes visible. These facts are necessarily fatal to the theories of Ploucquet, Clutterbuck, and Broussais. In the third place, it appears very probable, and almost evident, that the incipient movements in fever, that is, the first train of effects resulting from the application of the *ab externo* cause, are of a nature nearly the reverse of inflammation—namely, a weakened and disturbed state of the nervous and vascular systems, sometimes destructive of life before inflammation commences. In the fourth place, we have clear evidence that this first train of symptoms is succeeded, after a longer or shorter time, by turgescence and then inflammation of one or more organs, which inflammation and its consequences, effusion, adhesion, disorganization, &c. are very generally the cause of death, where the case proves fatal. From this view of the subject it is abundantly evident, (and actual practice every day confirms it,) that any plan of prevention or treatment founded on the exclusive doctrine of local inflammation being the source of fever, must be not merely ineffectual, but actually injurious. During the continuance of that train of phenomena which directly succeeds or is produced by the operation of the external cause of fever, the depletory treatment would be death. This depletion, however, is our main hope in the reaction which succeeds these incipient movements, as preventive of those fatal consequences of inflammation so strongly depicted by Dr. Jackson and all accurate observers. As for the inflammation, whether primary or secondary, having an exclusive site, in brain, stomach, or intestines, the theory is untenable, and totally inapplicable to practice.

The fifth chapter of the work before us is on prognosis, and contains a fund of the most valuable information and important remarks. We can make no pretence to a regular analysis of this, or any chapter in the book. Indeed, if all medical works were like those of Dr. Jackson, analytical reviews would be rendered nearly useless. We shall merely glance at a few prominent features of this chapter.

The fever of Andalusia commencing suddenly with intense head-ache, and gastric irritability simultaneous, indicated great danger. The same may be said where convulsions, stupor-like apoplexy, or outrageous delirium, with wild, agitated, bloated countenance, ushered in the fever. A rough dry tongue, a torpid statue-like aspect of countenance, a glistening glassy eye, a nauseous odour from the body, insensibility of the skin to blisters or sinapisms, dryness and paleness of the lips, suspension of cutaneous secretion, streaks or patches of a livid, green, or violet colour, are all indicative of the greatest danger. In respect to the pulse, our able author observed that when it became calm, regular, full, soft, and slow, at a certain point in the diseased course, unaccompanied with decided signs of favourable crisis, it was an almost certain sign of a fatal termination. As to animal temperature, a strong, deep, and concentrated impression of heat indicates danger; while a sharp, pungent, or caustic heat, accompanied with an arid, parched, and constricted skin, dry and shrivelled lips, &c. was a decidedly bad sign, though not invariably a mortal one.

Our author considers the yellow fever of the West Indies, though a dangerous disease, and much more rapid in its course than that of Andalusia, yet much less treacherous in its character than the latter, which is often masked and insidious. For a great many curious and important particulars respecting diagnostics, in the graver forms of fever, we refer to the work itself.

The sixth chapter is on the TREATMENT of the different forms of fever described in the preceding pages. To this, as to the other chapters, we cannot do justice in this analysis; but we hope our readers have already appreciated the value of the original too well, by the matter laid before them, to fail in perusing it, in propria forma, as a very valuable contribution to pathology and therapeutics. We shall still, however, endeavour to present some faint glimpse of this division of the work.

The first section of this chapter takes a rapid view of the Spanish practice in the epidemic fever in question. Arèjula, a physician of learning and accomplishment, but imbued with Brunonian principles, stands at the head of those who have written on this disease. He, somewhat in contravention of his own general principle, recommends a gentle emetic at the commencement, and as soon as the sense of coldness and shivering subsides, especially if the tongue be moist. If perspiration follow the operation of the emetic, a cup-full of tincture of bark is given every two or three hours. The treatment is often, it is said, successful, when the disease is mild. But if the prostration of strength be great at this period, Peruvian bark is given without loss of time, alternated with beef-tea or bouillon, and a glass of wine. The cinchona is ordered to be given every third hour, and bouillon the same; so that the patient has only one hour and a half of respite at a time from swallowing medicine or nourishment. Arèjula increases the quantity of bark by a scruple at a time, until the dose amounts to two or three drachms. Where the stomach is irritable, syrup of poppies, or opium and ether, are added to the bark; and where the bark cannot be retained in substance, the tincture is substituted in its place, with the addition of vitriolic ether, &c. Blisters and sinapisms are applied to different parts of the body, and removed as soon as they excite pain and irritation.

Such is the basis of Arèjula's practice. He asserts that his plan was successful, but furnishes us with no precise document by which we are enabled to judge. The idea of debility predominated in his mind—blood-letting is branded by him as directly destructive of life.

Dr. Flores de Moreno is physician to the king, and has the greatest practice in Cadiz. Having visited warm climates, and observed the English and American modes of treating yellow fever, he administers calomel and jalap, or calomel alone, every two hours till stools are procured, giving beef-tea in the intervals, by way of support; and accelerating the operation of the calomel by glysters of sea-water with some olive oil. Flores seldom fails, he asserts, to procure abundant bilious evacuations by these means; and, with bilious stools he usually obtains relief from the pressure of the more urgent symptoms of the disease. This step being secured, tincture of bark is given as preventive of the untoward accidents. Where symptoms of a threatening aspect, viz. heat, distress, and anguish at stomach, make their appearance on the third or fourth day, Dr. Flores directs a strong sinapism or blister to be applied to the pit of the stomach, and saline purgative enemata to be thrown up every three or four hours. Finally, he gives one or two spoonsfull, occasionally, of a mixture composed of balm-tea, Hoffman's liquor, and laudanum. Flores endeavours to uphold the patient's strength by bouillon, panada, rice cream, &c.

"He boasts success with some confidence; and his plan of treatment, it must be admitted, is better calculated to attain it than that of Dr. Arèjula; but it is by no means to be regarded as an efficient plan of treatment for the cure of the more aggravated forms of the yellow fever." 135.

We may here remark that both plans of treatment, if they be not decidedly and directly injurious, must be con sidered as fatal to the doctrine of Broussais, which makes fever the effect of local phlegmasiæ in the mucous membrane of the stomach and bowels. Should this theory be correct, it is scarcely possible that a single person could escape destruction, by either modes of treatment. At the same time we are far from believing that either modes of treatment were judicious; on the contrary, we agree with Dr. Jackson in thinking them any thing but efficient.

It may here be noticed, that a circumstance occurred, under our author's observation at Cadiz, in 1820, which proves that the utter proscription of venesection in this fever is not founded on a proper base.

"An English gentleman, a medical man, resident in that city, was attacked with the epidemic in one of its worst forms. He was seen, at an early period after the attack, by the writer and another English medical officer, and bled largely; that is, to between four and five pounds. Instead of being buried in two days, as some in the faculty had predicted, he appeared in the street in ten days in perfect health. He recovered and treated some persons who came under his own care in a similar manner as he had himself been treated. Not one of them died, and the most of them were fit for their duty in ten or twelve days." 136.

The novelty and severity of this discipline attracted the notice of the Spanish people, and bleeding and other means of depletion were employed soon afterwards by the most eminent practitioners of the city. The results were said to be favourable; and the yellow fever appeared, from the official reports, to be less fatal after this change in the method of treatment.

The mortality of this disease must have been prodigiously great, since, according to official returns, they lost 70 per cent at Xerez in 1820!

The Spanish physicians, according to our author, have what may be called a scholastic education. The name of Hippocrates or Galen weighs more in the balance than the evidence of their own senses. They have information but not knowledge. They read and treasure in their memory what they read. They rely on precedent and authority, but do not exercise their own reflective faculty on what they hear or see.

"The physician is little honoured any where in Europe, for his profession gives him neither power nor riches, nor influence in ruling the people; in Spain he is degraded to the condition of a menial. Few have courage to think, and no one dares to speak what he thinks, if it be contrary to the political views and prejudices of the authorities. A physician so circumstanced is of no positive value as a physician and man of science; and, as not conscious of value in himself, he becomes indifferent to the exercise of his duty, makes a short and superficial visit for a fee of four reals, collects as many reals as he can, and leaves the concerns of the patient very much to their own course. It thus happens in Spain, as in many other countries, that the first of human sciences as estimated by its merits, is converted, by a bigotted and overbearing policy, into a mean and prostituted trade—of some convenience, but of little real utility to mankind. 139.

Dr. Jackson must surely be aware that Great Britain, at least, ought to be exempted from "many other countries," where the science of medicine is converted by an overbearing policy into a prostituted trade. The faculty here are surely as free as the air they breathe; and however a few may be found depraved enough to prostitute the science itself and their own talents to a mean mercenary trading principle, yet we will venture to assert that, in the medical profession in this country, taking it collectively, there is more genuine philanthropy, disinterestedness, and humanity, than in all the other learned professions together. It is unfortunately too true that medical men permit themselves to fall into little paltry bickerings, jealousies, and scandals, as far as regards one another; but all things considered, there is a general and a glowing zeal in relieving human affliction wherever it exists, totally uninfluenced by the hope of reward. Nay, we will venture to assert still farther, that the eleemosynary patient is attended by the first talent in the land with as much anxiety for the event, as is the nobleman with his princely remuneration at command.

But to return. Dr. Jackson, in the second section of this chapter, details his own method of treating yellow fever, which he considers under three heads—namely, as abandoned to its own course—as interrupted, but not arrested—and as perfectly arrested by active means. The first plan is very common among Spanish physicians. The yellow fever, though not distinctly periodic in its movements, has yet a disposition to change, abate, or terminate at given times. Now if the disease remit in this way, without having previously disorganized some viscus or structure essential to life, the sensibility to common impressions is found to be greatly increased, from preceding suppression, and the healthy actions are readily reproduced by the common stimulants. This happens occasionally every where, and very often in Spain, where the people are mostly of sound constitutions, and a firm temper under affliction.

The second rule of practice in febrile diseases is that most commonly adopted, even by what are called active practitioners. But it cannot, of course, be considered as more than an auxiliary. It is directed against symptoms threatening particular organs. It mitigates violence, but it does not break the chain of the diseased movement, and thus arrest what is wrong.

The third or last is decisive, but difficult of execution, and requiring judgment and boldness to apply it at an early period of the disease. If applied, our author asserts, within six or eight hours from the commencement of indisposition, "it rarely fails to arrest or suspend the ostensible febrile act." Yet even in this case, perfect restoration of health is not always the immediate consequence of the suspension. It often requires three, five, or seven days to bring things to their natural state.

The Andalusian fever, Dr. Jackson thinks, is not an indomitable, though a dangerous disease. Its treatment requires to be modified, but the basis of it is the same in all cases. The first point is to arrest the diseased act—the second, to reproduce the action of health. As the impulse of the circulating blood sustains all the organic actions of the system, so the subtraction of it necessarily brings those acts to a pause, whether healthy or diseased. And as the atmosphere appears to be the general stimulant of animal life, so pure air, and especially movement through pure air, demonstratively maintains life in the greatest activity. "The impulse of the pure air to the proper vital organs is obviously the cause which reproduces the healthy act thus violently disturbed by the impulse of causes of morbific quality."

"The free admission of pure air into the sick apartment, even the transport of the sick through open air in convenient vehicles, is therefore primary and important among the means of remedy which are to be called into use for the renewal and support of health, as soon as the diseased act is brought to a pause of rest by artificial means. The application of cold water to the surface by aspersion or effusion, as more impressive by its weight and impulse than the ordinary breezes of the atmosphere, is still more powerful in its act; but it is perhaps less safe, and its effect cannot be so long sustained without inconvenience. When the movements of health are reproduced; various means, besides the impulses of the pure air of the atmosphere and occasional ablutions of cold water, conduce to maintain them in the forward course with more or less efficiency, that is, to prevent the recurrence of the disease in its original form." 143.

The above constitutes, according to our author, the basis of the medical treatment, in fevers similar to that of Andalusia, and this he would recommend in the said fever. He had an opportunity of observing the progress of the Andalusian fever in the Hospital San Juan de Dios, where little was done to oppose its natural course. He saw it treated and opposed vigorously at other places in Spain with good effect. In some cases, however, where vigour was used, success did not follow, "either owing to the want of necessary auxiliaries, or to hidden treachery in the character of the disease itself, which, as not easily seen, was not easily averted." The Andalusian fever, however, like that of the West Indies, varied into three principal forms, according as it acted on the sanguineous, lympho-phlegmatic, or serous temperament, requiring, of course, a modified treatment. These modifications are drawn by our author with the hand of a master, and are particularly recommended to the perusal of the medical practitioner, as containing excellent prinsiples for guidance in many other fevers than those of Andalusia or the tropics. We shall merely cull out a few particulars from this portion of the volume, hoping that they may excite to a more intimate acquaintance with the original.

Speaking of the second form of the fever, and alluding to the gastric irritability which is so very troublesome a symptom, he acknowledges that the prejudices against emetics is not unreasonable, since they are rarely useful, and often dangerous, especially if exhibited prior to abstraction of blood.

"But after the preparation of a given condition by bleeding or other means, the effect of emetics, particularly of emetics of white or blue vitriol, instead of being injurious, appeared to the writer to be eminently useful in allaying irritation, and in giving such a tone of vigour to the parts excited into action as precluded the chances of inflammation rising to excess. The power of the absorbent system is evidently diminished in the form of yellow fever now under consideration; and, as the action of the emetic has a tendency to increase absorption, and the action of emetics of turbith mineral a stronger tendency than others, a trial was made of it at Xerez in the year 1820,—and with signal good effect:—the person was convalescent in twenty-four hours. From this success, Mr. O'Halloran was induced to try it in others; and, from his experience, he considers it to be a remedy of great value—next in power perhaps to the lancet." 158.

A purgative which our author has found very useful in the West India fever consists of tincture of myrrh and aloes, with one drachm of æther, or an ounce of the rectified oil of turpentine, preceded by five or six grains of calomel, and three or four of genuine James's powder. The tincture produces fæculent stools more efficiently than any other form of purgative; but as it is slow in its operation, the æther or turpentine stimulates and gives it activity.

We find our limits so far overstepped that we must conclude this article with the following extract :—

"There is another point connected with the treatment of this disease at the period stated, which is of great importance to be studied and rightly understood. The head is often principally, at least prominently affected in this form of the yellow fever; and, as the head is the most important part in the animal body, the part connected with every function of the machine, the neglect of its diseased condition often compromises the life of the patient. Besides fits, convulsions, or deep stupor at the period of invasion, a certain degree of stupor—a peculiar degree of indifference and apathy, heaviness, fixity, or vacancy of the eye and countenance indicate distinctly that the substance of the brain is materially implicated in the morbid act. It is customary in such case, after bathing and bleeding, to cut off the hair, to shave the head, and to apply blisters to the scalp and nape of the neck. It is proper to do so; but it is reasonable to believe that the purpose would be attained with more certainty, if moxa or actual cautery were applied to the nape of the neck, as near as possible to the joining of the cranium with the vertebral pile. If the brain be in a state of torpor from what may be called congestion or pressure, the powerful stimulus from the heat of the cautery or moxa may be supposed to excite it into activity: if the structure be violated, no calculation can be made of a favourable result; but the physician may have cause to be satisfied that he has gone as far to attain it as human art, or human courage allow him to go." P. 174.

The last chapter in the work is on the law of quarantine; and we are sorry we cannot possibly spare room for any notice of his important remarks. We trust that even this very imperfect sketch of Dr. Jackson's work will lead many to consult it who might not otherwise be aware of the nature of its contents. We think that the venerable and estimable author has clothed the work before us in language which is entirely unobjectionable, and almost wholly divested of certain obscure and peculiar expressions or terms that somewhat disfigured his other productions. In fine, we hesitate not to state it as our belief, that, as the work *must* stand a lasting and honorable monument of Dr. Jackson's unparalleled zeal in the investigation of fever, so will it afford the most convincing proof of the success with which he has cultivated that great and important branch of medical science.

